# Engineering NAD^+^ availability for *Escherichia coli* whole-cell biocatalysis: a case study for dihydroxyacetone production

**DOI:** 10.1186/1475-2859-12-103

**Published:** 2013-11-09

**Authors:** Yongjin J Zhou, Wei Yang, Lei Wang, Zhiwei Zhu, Sufang Zhang, Zongbao K Zhao

**Affiliations:** 1 Division of Biotechnology, Dalian Institute of Chemical Physics, CAS, Dalian 116023, China; 2 Dalian National Laboratory for Clean Energy, Dalian Institute of Chemical Physics, CAS, Dalian 116023, China; 3 State Key Laboratory of Catalysis, Dalian Institute of Chemical Physics, CAS, Dalian 116023, China; 4 Present address: Department of Chemical and Biological Engineering, Chalmers University of Technology, Kemivägen 10, SE-412 96 Gothenburg, Sweden

**Keywords:** Cofactor engineering, NAD(H) level, NAD^+^ transporter, *Escherichia coli*, Dihydroxyacetone, Whole-cell biocatalysis

## Abstract

**Background:**

Whole-cell redox biocatalysis has been intensively explored for the production of valuable compounds because excellent selectivity is routinely achieved. Although the cellular cofactor level, redox state and the corresponding enzymatic activity are expected to have major effects on the performance of the biocatalysts, our ability remains limited to predict the outcome upon variation of those factors as well as the relationship among them.

**Results:**

In order to investigate the effects of cofactor availability on whole-cell redox biocatalysis, we devised recombinant *Escherichia coli* strains for the production of dihydroxyacetone (DHA) catalyzed by the NAD^+^-dependent glycerol dehydrogenase (GldA). In this model system, a water-forming NAD^+^ oxidase (NOX) and a NAD^+^ transporter (NTT4) were also co-expressed for cofactor regeneration and extracellular NAD^+^ uptake, respectively. We found that cellular cofactor level, NAD^+^/NADH ratio and NOX activity were not only strain-dependent, but also growth condition-dependent, leading to significant differences in specific DHA titer among different whole-cell biocatalysts. The host *E. coli* DH5α had the highest DHA specific titer of 0.81 g/g_DCW_ with the highest NAD^+^/NADH ratio of 6.7 and NOX activity of 3900 U. The biocatalyst had a higher activity when induced with IPTG at 37°C for 8 h compared with those at 30°C for 8 h and 18 h. When cells were transformed with the *ntt4* gene, feeding NAD^+^ during the cell culture stage increased cellular NAD(H) level by 1.44 fold and DHA specific titer by 1.58 fold to 2.13 g/g_DCW_. Supplementing NAD^+^ during the biotransformation stage was also beneficial to cellular NAD(H) level and DHA production, and the highest DHA productivity reached 0.76 g/g_DCW_/h. Cellular NAD(H) level, NAD^+^/NADH ratio, and NOX and GldA activity dropped over time during the biotransformation process.

**Conclusions:**

High NAD^+^/NADH ratio driving by NOX was very important for DHA production. Once cofactor was efficiently cycled, high cellular NAD(H) level was also beneficial for whole-cell redox biocatalysis. Our results indicated that NAD^+^ transporter could be applied to manipulate redox cofactor level for biocatalysis. Moreover, we suggested that genetically designed redox transformation should be carefully profiled for further optimizing whole-cell biocatalysis.

## Background

Cofactor-dependent redox biocatalysis has been shown as a powerful strategy for the production of valuable chemicals that are otherwise difficult to be synthesized [1,2]. Whole cells are preferred for industrial application because cofactors can be regenerated more efficiently [1]. To drive the redox chemistry to a specified direction, it is essential to manipulate intracellular redox state as well as cofactor levels [3]. Thus, various strategies have been applied to control the cofactor regeneration system or balance the enzyme activities of redox reactions. For example, H_2_O-forming NADH oxidase (NOX) has been applied for cofactor regeneration by engineered whole-cell biocatalyst for chiral compound production [4]. The intracellular cofactor concentration was also important to attain high efficiency especially in the case that the redox enzymes had high apparent *K*_m_ values to the cofactor [5]. Previous reports showed that exogenous supplied cofactors could improve the reaction rates under whole-cell catalysis conditions, although cells were permeated and external cofactor concentrations were applied at concentrations of over 0.5 mM [6-9]. We recently found that the nucleotide transporter NTT4 encoded by the *ntt4* gene from the chlamydial endosymbiont *Protochlamydia amoebophila* UWE25 [10] could enable *Escherichia coli* cells to uptake NAD(H) from the culture broth [11]. The NAD^+^ auxotrophic *E. coli* YJE003 cells expressing NTT4 cultivated in the media containing 40 μM NAD^+^ could realize the intracellular NAD(H) pool of 5.1 mM, which was 5.8-fold more than that of the wild-type cells [11]. We reasoned that such a unique NAD(H) supplementation system could be further explored to drive cellular redox chemistry.

**Figure 1:**
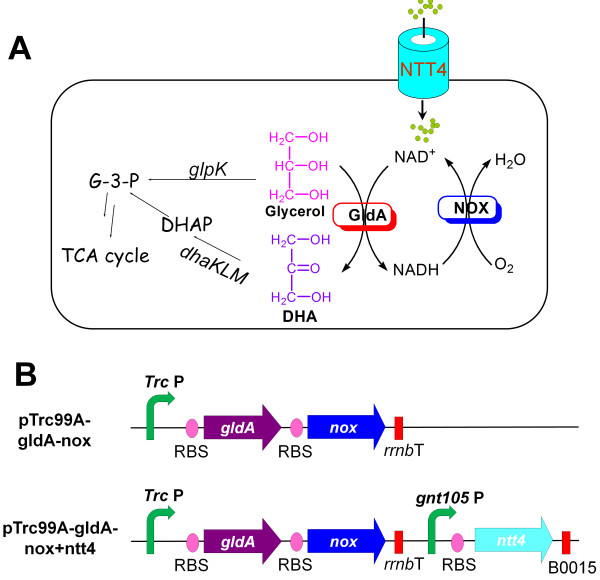
**Construction of recombinant *E. coli *strains for DHA production. (A)** Schematic representation of the engineered *E. coli* for DHA production by oxidizing glycerol with NAD^+^ regeneration and uptaking system; **(B)** Genetic arrangement of plasmids used for DHA production. *Trc* P, *Trc* promoter; *gnt105* P, gluconate transporter promoter 105 mutation; RBS, ribosome binding site; *rrnB* T, *rrnB* terminator; B0015, synthetic artificial terminator B0015 from IGEM.

Glycerol has become an inexpensive and readily available commodity as a byproduct of biodiesel industry, which makes it attractive to convert glycerol into higher-value products, such as 1,3-propanediol [12], glyceric acid [13], and dihydroxyacetone (DHA) [14]. Besides the clinical and biological applications, DHA is widely used as building blocks in chemical industry and as an artificial suntan in cosmetic industry [15]. *Gluconobacter oxydans* has been shown as an excellent DHA producer by the oxidation of glycerol using the membrane-bound pyrroloquinoline quinone-dependent glycerol dehydrogenase (GDH) [16]. Native GDH contains two subunits SldA and SldB and their membrane-associated feature may cause problems for heterologous expression. In *E. coli*, the *gldA* gene (ID: 6058353) encodes an endogenous NAD^+^-dependent GDH, which catalyzes reversible reactions for the interconversion of glycerol and DHA [17]. However, GldA has higher affinity and specificity (*K*_m (DHA)_ = 0.3 mM vs. *K*_m__(Glycerol)_ = 56 mM) towards DHA under normal physiological conditions, such that the conversion from DHA to glycerol (*k*_cat_/*K*_m_ = 5.7 × 10^4^ M^-1^ s^-1^) by GldA is far more efficient than the reverse reaction of glycerol to DHA (*k*_cat_/*K*_m_ = 4.0 × 10^2^ M^-1^ s^-1^) [17]. Here, we explored the possibility to engineer the NAD^+^ availability to reverse the GldA reaction direction for DHA production. For this purpose, we constructed recombinant *E. coli* strains that co-expressed two genes *gldA* and *nox*, and three genes *gldA*, *nox* and *ntt4*, for the production of DHA (Figure 1). Using this system as a model, we show that cellular NAD^+^ availability could be manipulated by different strategies and that the overall DHA specific titer was influenced by enzyme activity, cellular NAD^+^/NADH ratio, as well as cellular cofactor level. Our results provided useful information for the design and evaluation of redox biocatalyst to produce value-added chemicals.

## Results

### Construction of plasmids for DHA production

As no discernible DHA was observed when GldA alone was overexpressed in *E. coli* whole cells (date not shown), we introduced NOX as well as the NAD^+^ transporter NTT4 to increase NAD^+^ availability. Thus, two plasmids were constructed for DHA production from glycerol (Figure 1). For the plasmid pTrc99A-gldA-nox, the *gldA* gene from *E. coli* and the *nox* gene from *Enterococcus faecalis* were cloned into the vector pTrc99A with ribosome binding sites (RBS), under the control of the Trc promoter and the lacI repressor (Figure 1B). An rrnbT transcription terminator was located downstream of *nox*. For the plasmid pTrc99A-gldA-nox + ntt4, the NTT4 expression cassette in which the *ntt4* gene from *P. amoebophila* UWE25 was regulated by the promoter *gntT105* P was constructed, and inserted into the pTrc99A-gldA-nox backbone downstream of the rrnBT terminator using the RF cloning strategy [18]. Both plasmids ensured the expression of GldA for the oxidation of glycerol to DHA and NOX for NAD^+^ regeneration. In the case of pTrc99A-gldA-nox + ntt4, NTT4 was expressed to enable NAD^+^ uptake.

### Strain dependence of DHA production

**Figure 2:**
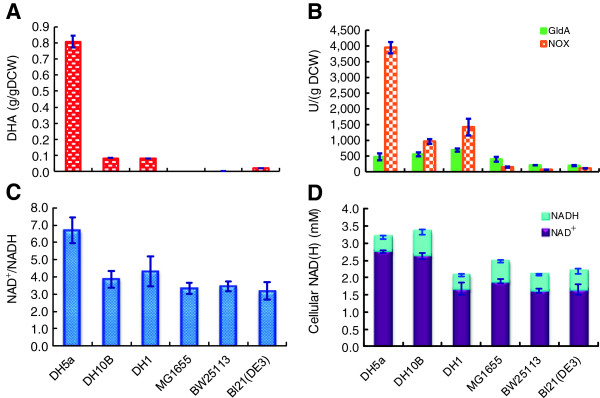
**DHA production by different *E. coli *strains harboring the plasmid pTrc99A-gldA-nox.** Cells were induced by IPTG for 20 h at 30°C, and collected for biotransformation experiments in 5 mL of reaction buffer with 20 g/L glycerol in 50-mL test tubes at 37°C, 200 rpm for 10 h. **(A)** DHA specific titer; **(B)** Initial enzymatic activity of GldA and NOX; **(C)** Initial NAD^+^/NADH ratio; **(D)** Initial intracellular NAD(H) level. The data represent the averages ± standard deviations (SDs) from three independent clones.

In *E. coli*, intracellular cofactor concentrations varied widely between different strains even under identical culture conditions in previous reports (Additional file 1: Table S1), which might affect the performance of whole-cell biocatalysts. Such phenomena may be used as a basis for host strain selection. A recent study showed that the efficiencies of different *E. coli* strains varied largely in the synthesis of (S)-1-(2-chlorophenyl)ethanol [19]. In addition, our previously constructed recombinant Bl21(DE3) whole-cells produced only 0.07 g/g_DCW_ DHA without external NAD^+^ supplementation [9]. Thus, we transformed pTrc99A-gldA-nox into 6 *E. coli* strains and investigated their biocatalysis profiles (Figure 2). DHA specific titers varied greatly among different hosts (Figure 2A). Recombinant *E. coli* DH5α had the highest DHA specific titer of 0.81 g/g_DCW_. Recombinant DH10B and DH1 had similar specific titer of 0.08 g/g_DCW_. However, when MG1655, BW2513 or BL21(DE3) was transformed with pTrc99A-gldA-nox, no DHA was detectable. Enzyme activity assay showed that NOX activity was well correlated with DHA titer (Figure 2B). While DH5α had a NOX activity of 3900 U/g_DCW_, DH10B and DH1 had much lower NOX activity. NOX activities for the other three strains were below 150 U/g_DCW_. A high NOX activity was beneficial for NAD^+^ regeneration, which was exemplified by NAD^+^/NADH ratios of different strains (Figure 2C). Indeed, DH5α, DH10B and DH1 had a relatively higher NAD^+^/NADH ratios, and the ratio reached 6.7 for DH5α. Intracellular NAD(H) levels were shown in Figure 2D. It was apparent that DH5α and DH10B had similar NAD(H) levels, and the other four strains had slightly lower yet similar values. Thus, high intracellular NAD(H) levels seemed necessary [20], but not sufficient for high DHA productivity. Instead, high initial NAD^+^/NADH ratio was well correlated with DHA titer (Figure 2A and C) and NOX activity was important to maintain high NAD^+^/NADH ratio. It remains puzzling that enzymatic activities, NAD^+^/NADH ratios and DHA productivities varied in different *E. coli* strains regardless of the presence of the same plasmid. However, similar phenomenon was reported recently that tyrosine production varied largely in 14 different *E. coli* strains containing the same pathway [21].

### Effects of cell growth on DHA production

**Figure 3:**
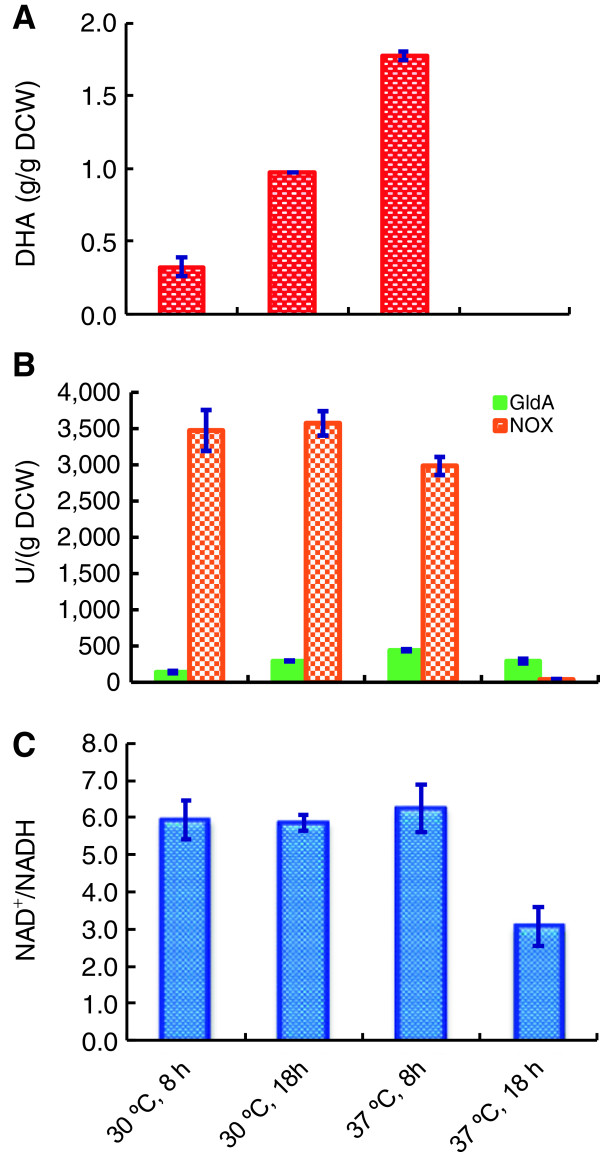
**DHA production by *E. coli *DH5α cells harboring the plasmid pTrc99A-gldA-nox.** Cells were cultivated at 30°C or 37°C for 8 h or 18 h after being induced by IPTG, and collected for biotransformation experiments in 5 mL of reaction buffer with 20 g/L glycerol at 37°C, 200 rpm for 10 h. **(A)** DHA specific titer; **(B)** Initial enzymatic activity of GldA and NOX; **(C)** Initial NAD^+^/NADH ratio. All data represent the averages ± SDs for three independent samples.

To test whether expression levels of GldA and NOX have an effect on DHA production, pTrc99A-gldA-nox transformed *E. coli* DH5α cells were cultivated under different conditions after being induced by IPTG. At 30°C, the DHA production was increased over time to 18 h. When the cell cultivated at 37°C, the DHA production was increased when the induction time extended from 4 h (0.62 g/g DCW) to 8 h, and dropped to below detection after 12 h (Figure 3A). So we chose the representative turning time point (8 h, 18 h) for enzyme activity and NAD(H) assay and investigating their correlation with DHA production. When cells were cultivated at 30°C for 8 h or 18 h after being induced by IPTG, intracellular NOX activities were similar. However, GldA activity of 290 U/g_DCW_ for the latter sample was 2.1-fold higher than that of the former (Figure 3B). As NAD^+^/NADH ratios for both samples were also similar (Figure 3C), a higher DHA specific titer of 0.97 g/g_DCW_ for the latter was likely resulted from a higher GldA activity. When cells were cultivated at 37°C for 8 h, the highest DHA specific titer of 1.78 g/g_DCW_ was obtained together with the highest GldA activity of 440 U/g_DCW_. The NAD^+^/NADH ratio for this sample was also slightly higher than that of the cells cultivated at 30°C. However, for the cells being cultivated at 37°C for 18 h, GldA activity, NOX activity and NAD^+^/NADH ratio were 290 U/g_DCW_, 39 U/g_DCW_, and 3.1, respectively, which were all substantially lower than those of other samples. As a result, DHA specific titer was only 0.01 g/g_DCW_. The reason for a reduced NOX activity might be due to the formation of protein inclusion bodies at a higher culture temperature, because NOX overexpression was toxic to the cell [22]. These results suggested that GldA determined the catalyst efficiency in the presence of adequate NOX for NAD^+^ regeneration. Although cells were harboring an identical plasmid, these results clearly demonstrated that protein expression levels could be significantly different under varied culture conditions, leading to drastic performance differences for whole-cell biocatalysis. Similar phenomena of culture conditions affecting biocatalyst efficiency have been observed in a recent study on L-glyceraldehyde production where glucose dehydrogenase was used for NAD^+^ regeneration [7].

### Promoter selection for NAD^+^ transporter expression

As has been demonstrated that intracellular NAD^+^ level and NAD^+^/NADH ratio had major effects on DHA production, we decided to further increase intracellular NAD^+^ level by NAD^+^ feeding. We [11] and others [10] showed that nucleotide transporter NTT4 could import NAD^+^ into *E. coli* cells. In this study, *ntt4* gene was cloned into vectors pBCTC and pBCTD, downstream constitutive promoters *gapA* P1 [23] and *gntT105* P [24], to give plasmids pBCTC-ntt4 and pBCTD-ntt4, respectively. Together with our previous construct pET15K-ntt4 in which T7 promoter was used [11], three promoters were tested. These three constructs and their corresponding backbone plasmids were transformed into *E. coli* DH5α cells. As shown in Table 1, when transformants were cultivated in the presence of 0.2 mM NAD^+^, pET15K-ntt4 led to 7.9% intracellular NAD^+^ level increment upon IPTG induction. Similarly, pBCTC-ntt4 led to 8.3% increment. However, pBCTD-ntt4 transformed cells accumulated NAD^+^ to 4.47 mM, which was 2.41-fold higher than that of pBCTD transformed cells. These results suggested that the promoter *gntT105* P was by far the most effective one in terms of NTT4 expression for NAD^+^ uptake. It should be pointed out that NTT4 is a membrane protein such that a high NTT4 expression seems toxic to the hosts which inhibited the cell growth (Additional file 1: Figure S1).

**Table 1:** **Intracellular NAD(H) levels of *E. coli *** **DH5α cells**^**a **^**harboring NTT4 expression and corresponding empty plasmids**

**Plasmid**	**Promoter**	**Cellular NAD(H) level (mM)**^**b**^		**Increment**
		**Empty vector**	**NTT4 expression**	
pET15k	T7	1.72 ± 0.03	1.85 ± 0.01	7.9%
pBCTC	*gap* P1	1.96 ± 0.00	2.13 ± 0.01	8.3%
pBCTD	*gntT105* P	1.86 ± 0.05	4.47 ± 0.14	141.0%

^a^Cells were cultivated at 37°C for 12 h in LB media containing 0.2 mM NAD^+^, collected by centrifugation and washed twice with PBS buffer.

^b^All data represent the averages ± SDs for three independent samples.

### Enhancing NAD^+^ supply for DHA production by using a NAD^+^ transporter NTT4

**Figure 4:**
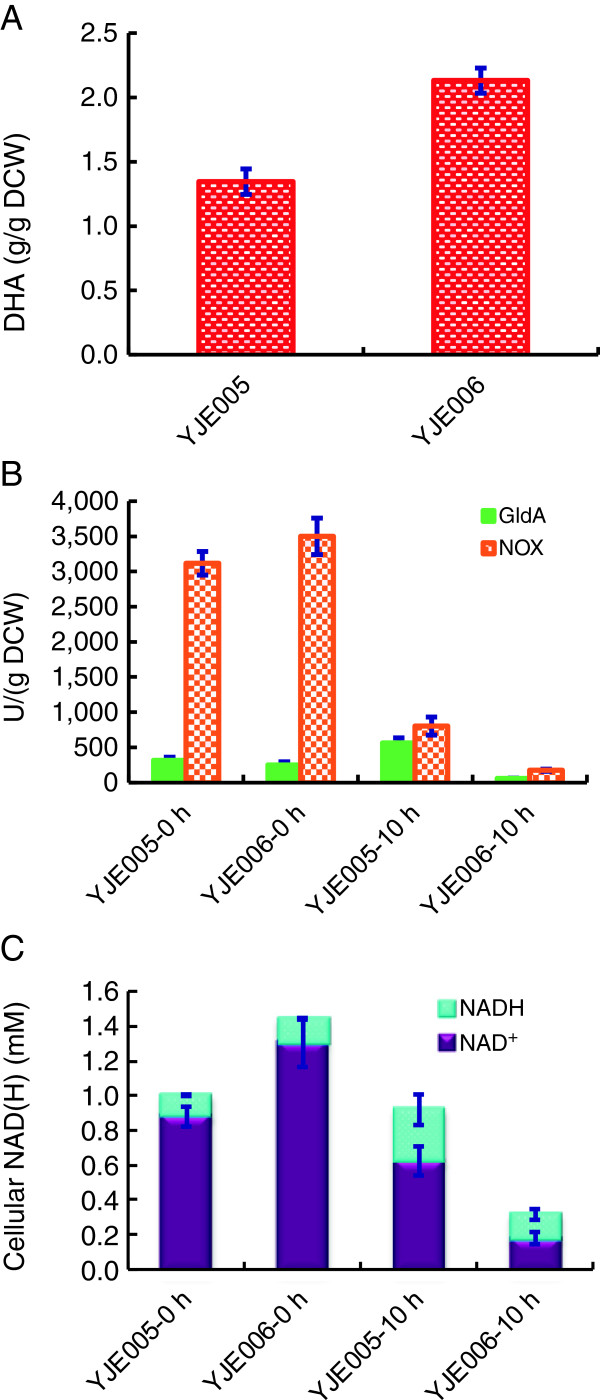
**DHA production by *E. coli *YJE005 and YJE006.** Cells were cultivated at 37°C for 8 h after being induced by IPTG, and collected for biotransformation experiments with 20 g/L glycerol and 0.2 mM NAD^+^ in 5 mL of reaction buffer in 50-mL test tubes at 37°C, 200 rpm for 10 h. **(A)** DHA specific titer; **(B)** Initial and end-point enzymatic activity of GldA and NOX; **(C)** Initial and end-point cellular NAD(H) level. All data represent the averages ± SDs for three independent clones.

*E. coli* DH5α cells were transformed with plasmids pTrc99A-gldA-nox and pTrc99A-gldA-nox + ntt4 to give strains YJE005 and YJE006, respectively. Both strains were cultivated at 37°C for 8 h in the presence of 0.2 mM NAD^+^, and whole-cell biocatalysts were prepared. DHA specific titer for YJE006 was 2.13 g/g_DCW_, which was 1.58-fold higher than that of YJE005 (Figure 4A), suggesting that NAD^+^ feeding during the cell culture stage was beneficial to DHA production. Initial GldA activity of YJE006 (250 U/g_DCW_) was slightly lower than that of YJE005 (310 U/g_DCW_), while initial NOX activity of YJE006 (3500 U/g_DCW_) was slightly higher than that of YJE005 (3100 U/g_DCW_). After being used for 10 h, both strains had significantly reduced NOX activities. While GldA activity of YJE006 dropped to 59 U/g_DCW_, GldA activity of YJE005, in sharp contrast, increased to 565 U/g_DCW_ (Figure 4B). Initial cellular NAD(H) level and NAD^+^/NADH ratio were 1.44 mM and 10.0 in YJE006, which were 1.44-fold and 1.34-fold higher than those of the YJE005, respectively (Figure 4C). Cellular NAD(H) level of YJE005 dropped by only 8% to 0.92 mM, but the NAD^+^/NADH ratio drastically dropped to 2.11 after 10 h. For YJE006, cellular NAD(H) level and the NAD^+^/NADH ratio were dropped by 78% and 87%, respectively, compared with those of the initial values. The fact that YJE006 cells had lower initial GldA activity but higher initial NOX activity, cellular NAD(H) level and NAD^+^/NADH ratio suggested that sufficient NAD^+^ supply and higher cellular redox state were more important than GldA activity to drive the system for DHA production. However, YJE006 cells appeared to lose enzyme activities and cellular cofactor more rapidly than YJE005 cells.

### DHA production under shake-flask conditions

**Figure 5:**
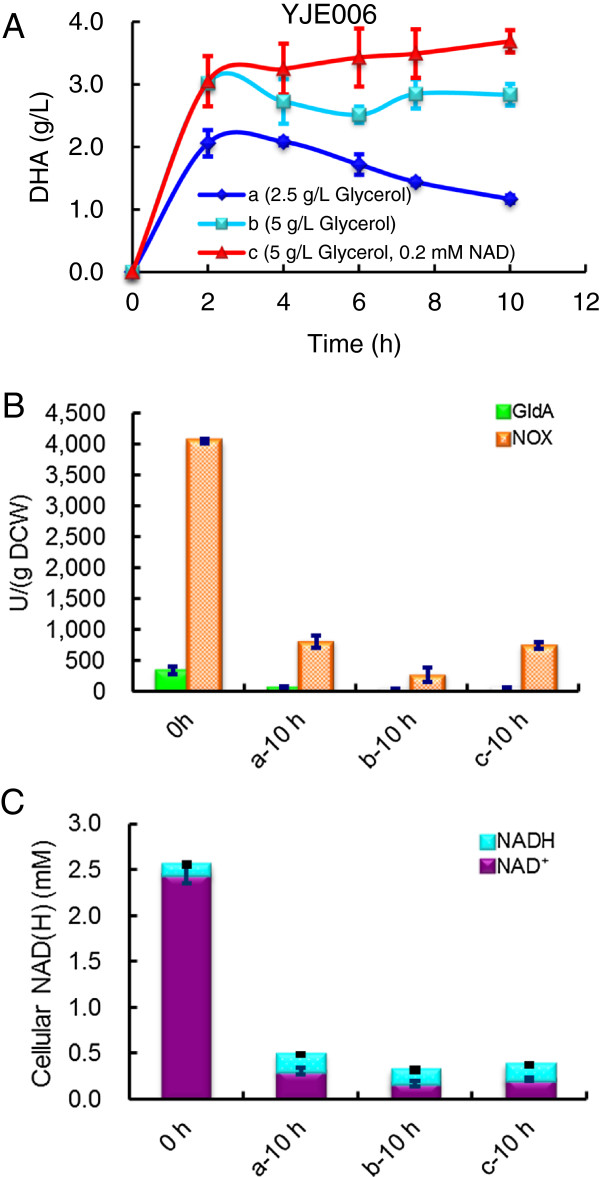
**DHA production by *E. coli *YJE006 whole cells in 20 mL of reaction buffer in 500-mL shake-flasks at 37°C, 200 rpm. (A)** The time course of DHA formation; **(B)** Initial and end-point enzymatic activity of GldA and NOX; **(C)** Initial and end-point cellular NAD(H) level. The data represent the averages standard deviations for three independent samples. All data represent the averages ± SDs for three independent samples.

In order to further investigate the NAD^+^ effect on the durability of the biocatalyst, the performance of YJE006 cells for DHA production was examined under shake-flask conditions after 2 h. When the reaction was initiated with 2.0 g_DCW_/L cells and 2.5 g/L glycerol, DHA concentration reached 2.08 g/L after 2 h (Figure 5A, Line a) and the biocatalyst activity reached 96 U/g_DCW_. When 5.0 g/L glycerol was used, the whole-cell catalyst produced 3.02 g/L DHA after 2 h with a higher biocatalyst activity of 140 U/g_DCW_ (Figure 5A, Line b). In both cases, DHA concentration dropped slowly over time, suggesting that DHA may be further consumed (Figure 1A). When the reaction was initiated with 2.0 g_DCW_/L and 5.0 g/L glycerol in the presence of 0.2 mM external NAD^+^, DHA production was burst to 3.05 g/L within 2 h, but it was slowly increasing for up to 10 h with a final titer of 3.69 g/L (Figure 5A, Line c). However, NAD^+^ supplementation failed to further improve the initial biocatalyst activity (141 U/g_DCW_). Again, activities of GldA and NOX dropped dramatically after 10 h (Figure 5B). However, cells had higher activities of GldA and NOX as well as NAD(H) level (b-10 h vs. c-10 h) after 10 h in the presence of external NAD^+^ supplementation. These results indicated NAD^+^ feeding at the biotransformation stage was also slightly beneficial to DHA production in NTT4 expressed recombinant *E. coli* cells.

## Discussion

Cellular redox state, roughly indicated by the NAD^+^/NADH ratio, is important for whole-cell redox biocatalysis. In *E. coli*, NAD^+^/NADH ratios ranging from 3 to 10 were previously documented [25]. It was expected that cellular NAD^+^/NADH ratio as well as NAD(H) level should have major effects on whole-cell biocatalysis. And co-expressing the *nox* gene encoding a H_2_O-forming NOX with GldA is expected to be helpful for reversing GldA activity toward DHA biosynthesis by efficient regenerating NAD^+^. Indeed, NOX has been widely used to enhance NAD^+^-dependent biosynthesis in both resting cells [4] and growing cells [26]. In this study, we found NOX activity was not only strain-dependent (Figure 2B), but also growth condition-dependent (Figure 3B), though cells were transformed with the same plasmid pTrc99A-gldA-nox. This may be attributed to the adaption ability difference against NOX expression among these strains because high NOX activity retarded cell growth and affected the metabolism [22]. Indeed, we also found that cell growth was negatively correlated with NOX activity (date not shown). Thus, while NOX expression provided driving force to maintain a higher NAD^+^ level and cellular NAD^+^/NADH ratio, it should be carefully tuned to avoid toxic effects on cellular physiology. It was found that DHA specific titer was positively correlated with the cellular NAD^+^/NADH ratio but not the cellular NAD(H) level (Figure 2). Similarly, introducing robust cofactor regeneration system increased the whole-cell biocatalysts efficiency [19,27]. However, enhancing the cellular NADP(H) pool alone without efficient cofactor regeneration failed to improve the redox biocatalyst efficiency [28]. Together, NAD^+^/NADH ratio driven by a cofactor regeneration system is more important than cellular cofactor level for an efficient oxidative bioreaction.

Once cofactor was efficiently regenerated, cellular cofactor level could also affect the biocatalyst efficiency [20]. External cofactor supplementation has been applied to increase intracellular NAD^+^ levels and NAD^+^/NADH ratios [6-8]. In those cases, cells were treated with permeating agents and external cofactor concentrations were high. We recently showed that NTT4 expression in *E. coli* led to uptaking NAD(H) from the culture broth [11]. Thus, *ntt4* was used to improve *E. coli* whole-cell biocatalytic efficiency by enhancing the cellular NAD(H) level. Actually, *ntt4* expression in YJE006 increased cellular NAD(H) level by 1.44 fold, indicating that NTT4 was functional in terms of uptaking external NAD^+^. As NTT4 could transport NADH efficiently [10] and increase the cellular reduction state with external NADH supplementation [11], it could also be used for increase the cellular NADH level for reduction biocatalyst such as asymmetric reduction of o-chloroacetophenone [8].

Although NTT4 expression strain YJE006 had a higher DHA production, it appeared to lose enzyme activities and cellular cofactor much more rapidly than YJE005. One possible reason was that NTT4 expression led to increased membrane permeability and other toxic effects. Therefore, it seemed challenging to maintain good NAD(H) durability for NTT4 expressions cells that were capable of uptaking external NAD^+^. As it requires alive cells to mediate continuous NAD^+^ transportation, NTT4 may have better potential profiting for NAD^+^-dependent multi-step biosynthesis in living cells such as 1-butanol production from glucose [29]. Although the NAD^+^ feeding strategy is cost prohibitive for bulk chemicals production, it may be useful for the production of high-value chemicals and pharmaceuticals. Alternatively, reconstruction of efficient heterologous NAD^+^ biosynthesis pathway [30] may increase cellular NAD(H) level for enhanced NAD(H)-dependent biosynthesis.

The highest specific DHA productivity reached 0.76 g/g_DCW_/h under shake-flask conditions for the first 2 h (Figure 5A), which was lower compared with other studies of more than 2 g/g_DCW_/h using *G. oxydans* as the hosts in small scale bioreactor [31], but much higher than that recombinant *Saccharomyces cerevisiae* of 0.03 g/g_DCW_/h from sugar [32]. The high DHA productivity of *G. oxydans* is attributed to the membrane-bound GDH, which can directly oxidize glycerol to DHA without material transfer across cell membrane [16]. However, our attempt, in reversing an endogenous cytosolic GldA catalysis for DHA production in recombinant *E. coli* by engineering NAD^+^ availability*,* provided some insights on optimizing whole-cell redox biocatalyst for other valuable chemicals and pharmaceuticals production.

As DHA concentration dropped over time after 2 h, it was possible that other metabolic steps consumed DHA. In this regard, disruption of the DHA and glycerol catabolic pathway related genes such as *dhaK* and *glpK* be useful (Figure 1A). Interestingly, external NAD^+^ supplementation during the whole-cell catalysis ensured a higher NOX activity and continuous accumulation of DHA for up to 10 h. These results indicated that NOX activity was beneficial to maintain oxidative ability of the NAD^+^-dependent whole-cell biocatalysis. It is worth mentioning that the initial specific biocatalyst activity (141 U/g_DCW_) was half to the initial GldA activity of 249 U/g_DCW_. This might be attributed to the substrate diffusion, product consumption, etc.

In summary, using oxidation of glycerol to DHA by recombinant *E. coli* whole-cells as a model system, we enhanced oxidation state and cellular cofactor level for increasing the catalytic efficiency by expressing NADH oxidase and NAD(H) transporter, respectively. As the overall biocatalytic performance is dependent upon the cellular cofactor level, redox state and the corresponding enzymatic activity, genetically designed redox transformation should be systematically profiled to identify optimal whole-cell biocatalysis.

## Material and methods

### Bacterial strains and plasmids

Cloning and plasmid propagation were performed with *E. coli* DH5α. The strains and plasmids are listed in Table 2. *E. coli* was routinely cultivated with agitation at 37°C or 30°C , 200 rpm in LB broth (10 g tryptone, 5 g yeast extract, 10 g NaCl per liter water) containing appropriate antibiotics (Kanamycin sulfate, 50 μg/mL; ampicillin, 100 μg/mL) if necessary.

**Table 2:** Strains and plasmids used in this study

**Strains or plasmids**	**Genotype or characteristic**	**Resources or references**
***E. coli *** **Strains**		
DH5α	*F-, φ80d/lacZ∆M15,* ∆*(lacZYA-argF)U169, deoR, recA1, endA1, hsdR17(rk-, mk+), phoA, supE44, λ-, thi-1, gyrA96, relA1*	TaKaRa
DH10B	F-,*mcr*A, ∆(*mrr*-*hsd*RMS-*mcr*BC), φ80*lac*Z∆M15, ∆*lac*X74, *rec*A1, *end*A1, *ara*D139, ∆ (*ara*, *leu*)7697, *gal*U, *gal*K, λ-, *rps*L, *nup*G/pMON14272/pMON7124	Invitrogen
DH1	F-, *glnV44*(AS), *λ*^*-*^, *rfbC1*, *gyrA96*(NalR), *recA1*, *endA1*, *thi-1*, *hsdR17*	CGSC
		(No. 6040)
MG1655	F-, *λ*^*-*^, *rph-1*	CGSC
		(No. 6300)
BW25113	*rrnB3,* ∆*lacZ4787, hsdR514,* ∆*(araBAD)567,* ∆*(rhaBAD)568 rph-1*	CGSC
		(No. 7636)
Bl21(DE3)	F–, *dcm, ompT, hsdS*(rB^–^, mB^–^), *gal,* λ(DE3)	Novagen
YJE005	DH5α/pTrc99A-gldA-nox	This study
YJE006	DH5α/pTrc99A-gldA-nox + ntt4	This study
**Plasmids**		
pMD18-T	*lacZ*, *pBR322 ori*, *bla*, cloning vector	TaKaRa
pTrc99A	*lacI, pBR322 ori, bla,* expression vector	Amersham Pharmacia
pET15K-ntt4	*ntt4* inserted within *Nde*I and *Bam*H I sites, *kan*	[11]
pBCTC-ntt4	*ntt4* inserted within *Sac* I and *Bam*H I sites, *kan*	This study
pBCTD-ntt4	*ntt4* inserted within *Sac* I and *Bam*H I sites, *kan*	This study
pTrc99A-gldA-nox	*gldA* and *nox* transcription under *Trc* promoter	This study
pTrc99A-gldA-nox + ntt4	*gldA* and *nox* transcription under Trc promoter, *ntt4* under gntT105p promoter.	This study

### Reagents

All primers used in this study (Table 3) were custom synthesized from Invitrogen (Shanghai, China) and DNA sequencing was performed in TaKaRa (Dalian, China). *Dpn* I, PrimeSTAR HS DNA polymerase and all other reagents for genetic manipulation were purchased from TaKaRa (Dalian, China). DNA gel purification kit and plasmid extraction kit were purchased from Beyotime (Haimen, China). All chemicals were purchased from Sigma (Shanghai, China).

**Table 3:** **Primers used in this study**^**a**^

**Primer**	**Sequence(5′-3′)**	**Function**
gldA-F0	TGCTGTATATAGCGCCGCACAAG	*gldA*_cloning
gldA-R0	AGGTTGGTATTGGCCTGGATTTG	
gldA-F1	CAATTTCACACAGGAAACAGACCATGGACCGCATTATTCAATCAC	*gldA*_amplification
gldA-R1	GTGTATATCTCCTTCTCTAGTAGCGATCTATTATTCCCACTCTTGCAGG	
Nox-F1	CTACTAGAGAAGGAGATATACACATGAAAGTCGTAGTCGTAGG	*nox*_amplification
Nox-R1	CAAAACAGCCAAGCTTGCATGCCTGCAGTTACATATTTTCTAAAGCGGCTTG	
gapAP1-F1	TCG**GATATC**GAGGCGAGTCAGTCGCGTAATGC	Promoter gapAP1 amplification
gapAP1-R1	ACC**GAATTC**GATCTCATATGTTCCACCAGCTATTTGTTAG	
gntT105P-F1	CCGTT**GATATC**TGAAAGGTGTGCGCGATCTCAC	PromotergntT105P amplification
gntT105P-R1	G**GAATTC**TATCTCCTTATTCATTTGCGCTGGGTAACGTCAATTT	
ntt4-F1	TTC**GAGCTC**ATGAGTAAAACAAACCAGG	
ntt4-R1	AGA**GGATCC**TTAGTGATGATGATGATGATGTTTTTTTATAAAAG	*ntt4* amplification
gntT105P-F2	CAAACTCTTTTTGTTTATTTTTCTAAATACATGAAAGGTGTGCGCGATCTC	gntT105P + ntt4 amplification
ntt4-R2	CGTTTTATTTGATGCCTGGATCCGCGTCGACTCTAGAGGATCC	
T-F1	GGATCCTCTAGAGTCGACGCGGATCCAGGCATCAAATAAAACG	Terminator BBa_B0015 amplification
T-R1	GTATTTAGAAAAATAAACATATAAACGCAGAAAGGCCCAC	

^a^The restriction sites were bolded and the ribosome binding sites (RBS) were underlined.

### DNA manipulation

The *gldA* (NCBI GeneID: 6058353) gene was amplified from *E. coli* DH5α genomic DNA using primer pair gldA-F0/gldA-R0 and cloned into pMD18T to give pMD18T-*gldA*. The *nox* (NCBI GeneID: 1200486) gene was amplified from pMD18T-*nox*, which was constructed by cloning the *nox* gene from *Enterococcus faecalis* (CGMCC 1.130) into the pMD18T. The *gldA-nox* co-expression cassette was constructed with the modified one-step overlap extension (SOE) PCR strategy described previously [33]. Briefly, *gldA* and *nox* were amplified with primer pairs gldA-F1/gldA-R1 and nox-F1/nox-R1, and purified *gldA* and *nox* fragments (molar ratio 1:1, about 200 ng each) were mixed. To the mixture were added 3 μL of dNTP (2.5 mM each), 5 μL 5 × PrimerStar buffer, 1.25 U PrimeSTAR HS DNA polymerase, and H_2_O to a total volume of 25 μL. PCR amplification was performed according to the thermocycle conditions of 95°C for 5 min, 15 cycles of 98°C for 10 s, 68°C for 3 min, and 68°C for 10 min. Next, 2 μL of unpurified PCR products was used as the template using the primer pair gldA-F1/nox-R1 for normal PCR amplification in a total volume of 100 μL. The purified *gldA-nox* cassette was cloned into the pTrc99A using the restriction-free (RF) cloning strategy [11] to give plasmid pTrc99A-gldA-nox.

The *ntt4* (NCBI GeneID: 2780098) gene containing the 3'-end 6 × His-tag encoding sequence was cloned from the vector pET15k-*ntt4*[11]. The constitutive glyceraldehyde-3-phosphate dehydrogenase promoter P1 *gapA* P1 [23] and the internal operator of gluconate transporter promoter 105 mutant *gntT105* P [24] was cloned from *E. coli* DH5α genomic DNA using primer pairs gapAP1-F/gapAP1-R and gntT105P-F1/gntT105P-R1, respectively. Then, *Eco*R V-*Eco*R I digested promoters were cloned into the *Eco*R V-*Eco*R I site of pTrc99A to substitute the Trc promoter, resulting in the constitutive expression vectors pBCTA and pBCTB. The function of these two vectors was checked by constitutive expression of red fluorescent protein (date not shown). Lastly, *ntt4* was cloned into the *Sac* I-*Bam*H I site locating downstream of the constitutive promoter of pBCTA and pBCTB after cloning with the primer pair ntt4-F1/ntt4-R1, and the *bla* was replaced by the *kan* using a RF cloning strategy, resulting in plasmids pBCTC-*ntt4* and pBCTD-*ntt4*, respectively.

The *ntt4* constitutive expression cassette was also constructed and cloned into pTrc99A-gldA-nox. The gntT105P-*ntt4* was amplified from the pBCTD-*ntt4* using the primer pair gntT105P-F2/ntt4-R2 and terminator B0015 cloned using the primer pair T-F1/T-R1 from the international genetically engineered machine competition (IGEM, http://partsregistry.org/Part:BBa_B0015). These two DNA fragments were fused with a modified SOE PCR approach (33), and cloned into pTrc99A-gldA-nox locating downstream of the rrnBT terminator, resulting in plasmid pTrc99A-gldA-nox + ntt4.

### Whole-cell biocatalyst preparation

Recombinant *E. coli* cells harboring appropriate plasmid were cultivated in LB medium supplemented with appropriate antibiotics at 37°C, 200 rpm, to the early exponential phase (OD_600_ = 0.2–0.4). Cultures were induced by adding IPTG to a final concentration of 0.1 mM (and 0.2 mM NAD^+^ if needed), and cultivated for additional 8 h or 18 h at appropriate temperature (37°C or 30°C), 200 rpm. Cells were harvested by centrifugation (2,000 *g*, 5 min) and washed twice with 0.1 M potassium phosphate buffer (pH 9.0).

### DHA production

As GldA had a higher activity toward glycerol dehydrogenation [34] and our previous study [9] showed DHA production reached the highest at pH 9.0. The IPTG induced *E. coli* cells were resuspended in 5 mL of 0.1 M potassium phosphate buffer (pH 9.0) for 10 h in 5 mL of potassium phosphate buffer containing 20 g/L glycerol in 50-mL test tubes; or in 20 mL of the buffer containing 2–5 g/L glycerol in 500-mL shake flasks. NAD^+^ was added into the reaction to a final concentration of 0.2 mM when necessary. All reactions were performed at 37°C, 200 rpm. All the data represent the averages standard deviations from at least three independent samples.

### DHA quantification

DHA was assayed according to a known method with minor modifications [35]. Briefly, biotransformation mixtures were centrifuged at 10,000 *g* for 2 min. Exactly 20 μL of supernatants were mixed with 180 μL diphenylamine reagent containing 1% (w/v) diphenylamine and 10% (v/v) sulfuric acid in acetic acid, and heated at 105°C for 20 min. Then, the absorbance at 620 nm were recorded after cooling to room temperature, and DHA concentrations were quantified according to a standard curve obtained under identical conditions.

### Enzyme activity assay

*E. coli* dry cell weight (DCW) was weight by converting OD_600_ value with a coefficient of 0.275 g_DCW_/(L × OD_600_), which was determined by freezer drying the *E. coli* cells according to our recently report [36]. As the DHA production was performed at 0.1 M potassium phosphate buffer (pH 9.0) due to GldA activity has higher activity as mentioned above, all enzymatic assays were performed at consistent pH of 9.0. About 2 × 10^9^*E. coli* cells were harvested, washed twice with 0.1 M potassium phosphate buffer (pH 9.0), and stored as cell pellets at -80°C. For enzyme assays, cell pellets were resuspended in 0.2 mL of lysis buffer (10 mM Tris-Cl, 1.0 mM MgCl_2_, 1 mg/mL lysozyme and 0.1 mg/mL DNase, pH 8.0) and incubated at 37°C for 30 min. GldA activity was estimated by recording the absorbance increase at 340 nm and assays were performed at 25°C, in 100 μL of 0.1 M potassium carbonate buffer (pH 9.0) containing 5 mM NAD^+^, 100 mM glycerol and 5 μL of crude cell lysates, which was with minor modifications from a previous report [37]. NOX activity was measured by recording the absorbance decrease at 340 nm and assays were performed at 25°C, in 100 μL of 0.1 M potassium phosphate buffer (pH 9.0) containing 0.4 mM NADH and 1 μL (if the cell NOX activity was more than 1000 U/g_DCW_) or 5 μL of (if the cell NOX activity was less than 1000 U/g_DCW_) crude cell lysates.

### Cofactor measurement

Cell pellets (containing about 2 × 10^9^ cells) were washed twice with 0.1 M potassium phosphate buffer, and then treated at 55°C for 10 min in 150 μl of 0.2 M NaOH (for NADH extraction) or 150 μl of 0.2 M HCl (for NAD^+^ extraction). The extracts were neutralized by adding 150 μl of 0.1 M HCl (for NADH extraction) or 150 μl of 0.1 M NaOH (for NAD^+^ extraction). The cellular debris was removed by centrifuging at 12,000 *g* for 5 min. Supernatants were transferred to new tubes and stored at -80°C until assay. NAD(H) was quantified using a sensitive enzymatic cycling assay as reported previously [11].

## Competing interests

The authors declare that they have no competing interests.

## Authors’ contributions

YJZ and ZKZ conceived and designed the experiments. YJZ and WY performed the experiments. YJZ, LW, ZZ, SZ and ZKZ analyzed the data. YJZ and ZKZ wrote the manuscript. All authors read and approved the final manuscript.

## Supplementary Material

Additional file 1 **Table S1.** Cellular NAD(H) level of different *E. coli* strains from literatures. **Figure S1.** NTT4 expression under *gntT105*P strongly retarded *E. coli* DH5α growth. Click here for file
