# Supplementary material for: Engineering NAD+ availability for Escherichia coli whole-cell biocatalysis: a case study for dihydroxyacetone production
Source: Microb Cell Fact. 2013 Nov 9;12:103. doi: 10.1186/1475-2859-12-103 (PMC3831814; doi:10.1186/1475-2859-12-103)
Supplement: Additional file 1 — Table S1. Cellular NAD(H) level of different E. coli strains from literatures. Figure S1. NTT4 expression under gntT105P strongly retarded E. coli DH5α growth. [file 1475-2859-12-103-S1.doc]

**Additional file 1**

Table S1. Cellular NAD(H) level of different *E. coli* strains from literatures.

| Strains | NAD(H)  (mM) | Cultivation condition | Assay method | References |
| --- | --- | --- | --- | --- |
| BW25113 | 0.9 | M9 media, shake flask | Enzymatic cycling |  |
| BW25113 | 1.4-1.7 | M9 media, shake flask | Enzymatic cycling |  |
| Bl21(DE3) | 0.6 | LB media, shake flask | Enzymatic cycling |  |
| MC4100 | 5.5-7.4^a^ | LB media, chemostat | Enzymatic cycling |  |
| DH10B | 8.8-19.0^a^ | LB media, chemostat | Enzymatic cycling |  |
| DH1 | 13.8-21.7^a^ | TB media, shake flask | Enzymatic cycling |  |
| MG1655 | 0.1^a^ | MOPS media, shake flask | LC-MS/MS |  |

a: All these NAD(H) level were given with the dry cell weight concentration (Cdcw (NADH)) in the literatures. So the NAD(H) volume concentrations (Cv(NADH)) were calculated from Cdcw (NADH) as below:


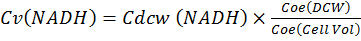


Cell weight coefficient Coe(DCW) is 0.275 gDCW/(L×OD600) determined in Material and Methods, and cell volume coefficient (Coe(Cell Vol)) is 10^-3^ L/(L×OD600) as described previously .


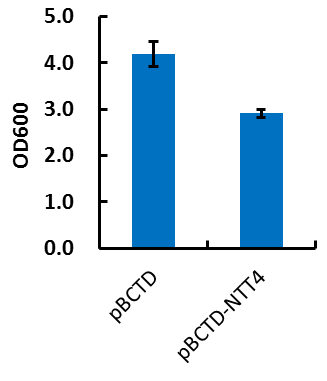


Figure S1. NTT4 expression under *gntT105*P strongly retarded *E. coli* DH5α growth.

Recombinant *E. coli* DH5α cells harboring appropriate plasmid were cultivated at 37 °C, 200 rpm for 12 h in 5 mL of LB medium supplemented with 50 µg/mL Kanamycin sulfate and 0.1 mM NAD^+^. Then the cells was collected for NAD(H) assay as described in Material and Methods.

1. Zhou YJ, Wang L, Yang F, Lin XP, Zhang SF, Zhao ZK: **Determining the extremes of the cellular NAD(H) level by using an *Escherichia coli* NAD^+^-auxotrophic mutant.** *Appl Environ Microbiol* 2011, **77:**6133―6140.

2. Dhamdhere G, Zgurskaya HI: **Metabolic shutdown in *Escherichia coli* cells lacking the outer membrane channel TolC.** *Mol Microbiol* 2010, **77:**743―754.

3. Yang W, Zhou YJ, Zhao ZK: **Production of dihydroxyacetone from glycerol by engineered *Escherichia coli* cells co-expressing *gldA* and *nox* genes.** *Afr J Biotechnol* 2013, **12:**4387―4392.

4. San KY, Bennett GN, Berrios-Rivera SJ, Vadali RV, Yang YT, Horton E, Rudolph FB, Sariyar B, Blackwood K: **Metabolic engineering through cofactor manipulation and its effects on metabolic flux redistribution in *Escherichia coli*.** *Metab Eng* 2002, **4:**182―192.

5. Bond-Watts BB, Bellerose RJ, Chang MC: **Enzyme mechanism as a kinetic control element for designing synthetic biofuel pathways.** *Nat Chem Biol* 2011, **7:**222-227.

6. Holm AK, Blank LM, Oldiges M, Schmid A, Solem C, Jensen PR, Vemuri GN: **Metabolic and transcriptional response to cofactor perturbations in *Escherichia coli*.** *J Biol Chem* 2010, **285:**17498-17506.
